# Correction: Afjei et al. A New Nrf2 Inhibitor Enhances Chemotherapeutic Effects in Glioblastoma Cells Carrying p53 Mutations. *Cancers* 2022, *14*, 6120

**DOI:** 10.3390/cancers17142408

**Published:** 2025-07-21

**Authors:** Rayhaneh Afjei, Negar Sadeghipour, Sukumar Uday Kumar, Mallesh Pandrala, Vineet Kumar, Sanjay V. Malhotra, Tarik F. Massoud, Ramasamy Paulmurugan

**Affiliations:** 1 Department of Radiology, Molecular Imaging Program at Stanford (MIPS), Canary Center at Stanford for Cancer Early Detection, Stanford University School of Medicine, 3155 Porter Drive, Palo Alto, CA 94305, USA; seyedehrayhanehafjei@gmail.com (R.A.); sadeghi@stanford.edu (N.S.); udkr8817@stanford.edu (S.U.K.); 2 Department of Radiation Oncology, Stanford University School of Medicine, 3155 Porter Drive, Palo Alto, CA 94305, USA; pandrala@ohsu.edu (M.P.); iamvineet@gmail.com (V.K.); malhotsa@ohsu.edu (S.V.M.); 3 Department of Cell, Development and Cancer Biology, Knight Cancer Institute, Oregon Health & Science University, Portland, OR 97201, USA; 4 Center for Experimental Therapeutics, Knight Cancer Institute, Oregon Health & Science University, Portland, OR 97201, USA

## Figure Legend

In the original publication [1], there was a mistake in the legend for Figure S2. The subfigure (D) in Figure S2 refers to Figure S3, not S2 as it was written before. The correct legend appears below:

Figure S2. Whole western blotting membranes to show the location of each band and the intensities. (A) Figure 3C in the main manuscript. (B) Figure 4D in the main manuscript. (C) Figure 4E in the main manuscript. (D) Figure S3 in the Supplemental.

## Error in Figure

In the original publication [1], there were mistakes in the arrangement and cropping of representative images from the original figures for Figures S2, S10 and S11.

The correct Figure S2, Figure S10 and Figure S11 with the respective modified legends appear below now. The authors apologize for any inconvenience caused and state that the scientific conclusions are unaffected. This correction was approved by the Academic Editor. The original publication has also been updated.

## Figures and Tables

**Figure S2:**
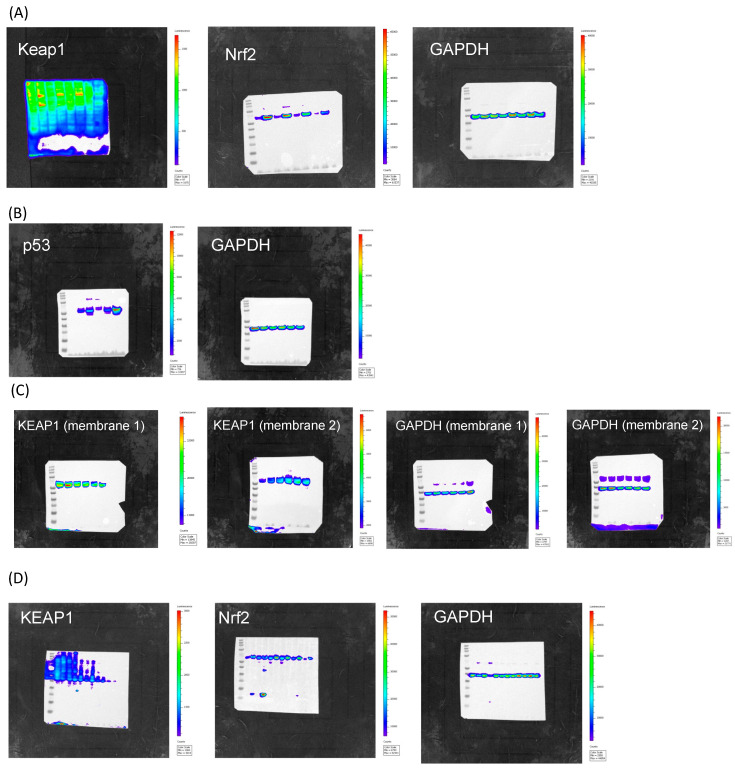
Whole western blotting membranes to show the location of each band and the intensities. (**A**) Figure 3C in the main manuscript. (**B**) Figure 4D in the main manuscript. (**C**) Figure 4E in the main manuscript. (**D**) Figure S3 in the Supplemental.

**Figure S10:**
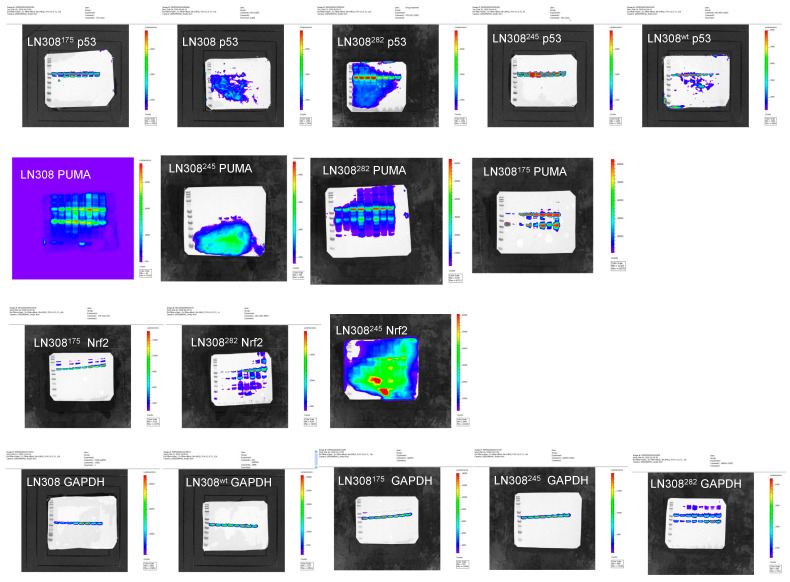
Whole western blotting membranes to show the location of each band and the intensities, for Figure 7 in the manuscript.

**Figure S11:**
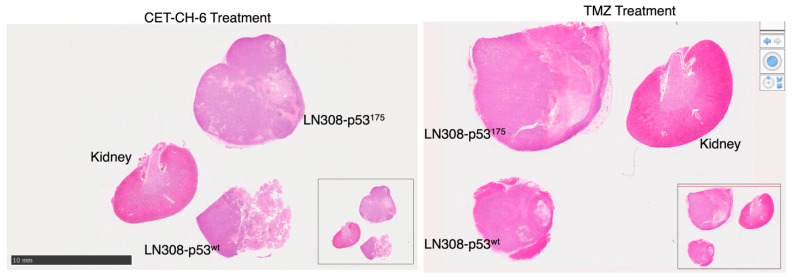

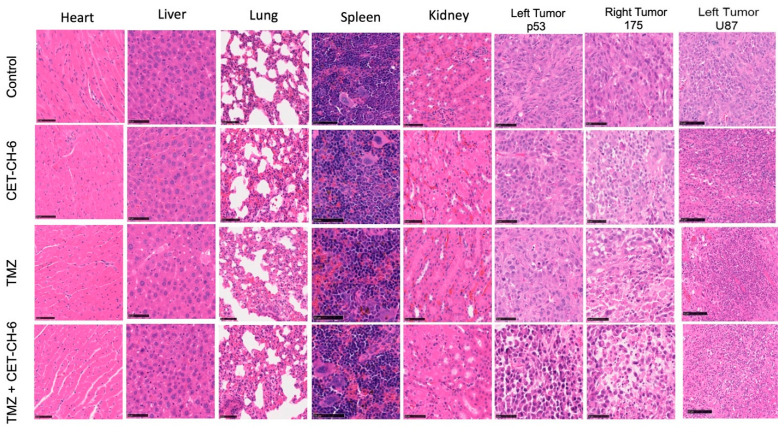
H&E staining of different organs and tumors. NSG mice bearing U87-MG, LN308-p53^175^ and U87-MG-p53^wt^ cells. The bars represent 50 μm.
