# Supplementary material for: A New Nrf2 Inhibitor Enhances Chemotherapeutic Effects in Glioblastoma Cells Carrying p53 Mutations
Source: Cancers (Basel). 2022 Dec 12;14(24):6120. doi: 10.3390/cancers14246120 (PMC9775980; doi:10.3390/cancers14246120)
Supplement: Supplementary file 1 [file cancers-14-06120-s001.zip › cancers-1750378-supplementary.pdf]

cancers

MDPI

Supplementary Material: A New Nrf2 Inhibitor Enhances Chemotherapeutic Effects in Glioblastoma Cells Carrying p53 Mutations

Rayhaneh Afjei, Negar Sadeghipour, Sukumar Uday Kumar, Mallesh Pandrala, Vineet Kumar, Sanjay V. Malhotra, Tarik F. Massoud * and Ramasamy Paulmurugan *

Supplementary Materials and Methods

General Methods

All the reagents and solvents were obtained at the highest commercial quality from sources such as Sigma-Aldrich and Fisher Scientific and were used without further purification. The reactions were monitored by TLC using pre-coted silica gel plates (Merck, silica gel 60 F_{254}). Flash chromatography was carried out using a CombiFlash Rf+ Lumen chromatography system (Teledyne ISCO, Lincon, NE, USA). ^{1}H (400 MHz) and ^{13}C (101 MHz) NMR spectra were recorded on an Agilent 400-MR NMR spectrometer, using appropriate deuterated solvents, as needed. Chemical shifts (δ) were reported in parts per million (ppm) upfield from tetramethylsilane (TMS) as an internal standard. Coupling constants (*J*) were reported in hertz (Hz), and s, br.s, d, t and m are designated as singlet, broad singlet, doublet, triplet and multiplet, respectively. LC-MS analysis was performed on an Agilent 6490 iFunnel Triple Quadrupole Mass Spectrometer from Agilent Technologies Inc. (Santa Clara, CA, USA). The mass spectrometer was operated under positive ionization mode. All tested compounds were >95% pure.

Synthesis of (E)-3-(3-(5-Hydroxy-2-Methoxyphenyl)-3-Oxoprop-1-en-1-yl)Benzonitrile (CET-CH-6)

The chalcone **CET-CH-6** was prepared according to the previously reported procedure for a similar analogue,[21] with several modifications. To a stirred solution of 1-(5-hydroxy-2-methoxyphenyl)ethan-1-one **1** (0.2 g, 1.2 mmol) in ethanol (10 mL) was added 10% NaOH solution in water (2 mL) and allowed to stir at rt for 5 -10 min. Then, 3-formylbenzonitrile**2** (0.15 g, 1.2 mmol) was added slowly. Upon completion of compound **2** addition, the reaction mixture color turned to pale yellow. The mixture was allowed to stir at rt until both of the starting materials disappeared on TLC. Water (10 mL) was added to the reaction mixture and neutralized it with 0.1 N HCl. The product was extracted with ethyl acetate (2 X 50 mL). The combined organic layer was washed with water followed by brine solution. The organic phase was dried over Na_2SO_4, filtered and evaporated to dryness. The crude material obtained was purified on a silica gel column using 0-30% ethyl acetate in hexane as an eluent. The pure fractions were combined and evaporated to dryness to afford the desired compound as a pale-yellow oil. The oil compound was chased with hexane to obtain the desired compound as a pale-yellow solid (0.15 g, 45 % yield). ^{1}H NMR (400 MHz, DMSO-d_6) δ 9.26 (s, 1H), 8.24 (t, *J* = 1.6 Hz, 1H), 8.09 – 8.03 (m, 1H), 7.86 (ddd, *J* = 7.7, 1.6, 1.1 Hz, 1H), 7.65 – 7.58 (m, 1H), 7.52 (d, *J* = 1.8 Hz, 2H), 7.02 (d, *J* = 8.8 Hz, 1H), 6.97 – 6.88 (m, 2H), 3.77 (s, 3H). ^{13}C NMR (101 MHz, DMSO- d_6) δ 192.0, 151.5, 151.5, 140.2, 136.5, 133.9, 133.2, 132.5, 130.6, 129.5, 129.4, 120.4, 118.9, 115.9, 114.5, 112.6, 56.8. LC-MS (ESI-Q): m/z found 280.0, calculated for [$C_{17}H_{13}NO_3$+H] 280.09.

Supplementary Figures

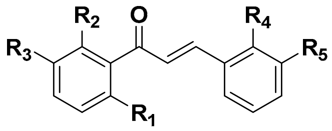

Compound	R_1	R_2	R_3	R_4	R_5
CET-CH-1	OCH_3	H	H	H	H
CET-CH-2	H	H	H	CF_3	H
CET-CH-3	OCH_3	H	H	CF_3	H
CET-CH-4	OCH_3	OCH_3	H	CF_3	H
CET- CH-5	OCH_3	H	H	H	CF_3
CET- CH-6	OCH_3	HH	OH	H	CN

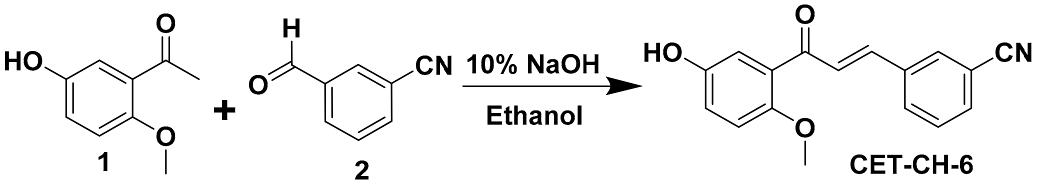

Figure S1. Schematic workflow showing the synthesis of CET-CH6.

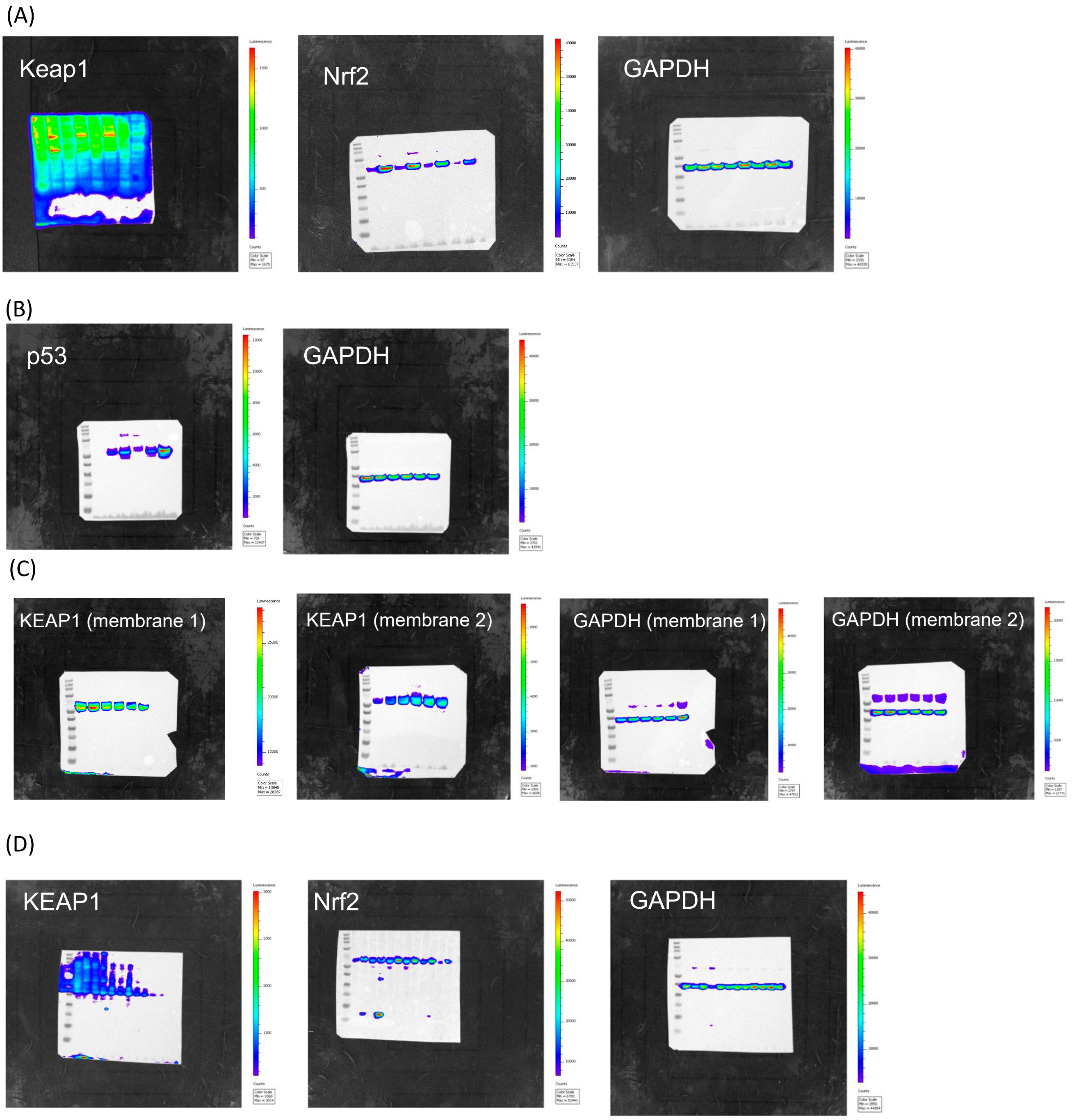

Figure S2. Whole western blotting membranes to show the location of each band and the intensities. (A) Figure 3C in the main manuscript. (B) Figure 4D in the main manuscript. (C) Figure 4E in the main manuscript. (D) Figure S3 in the supplemental.

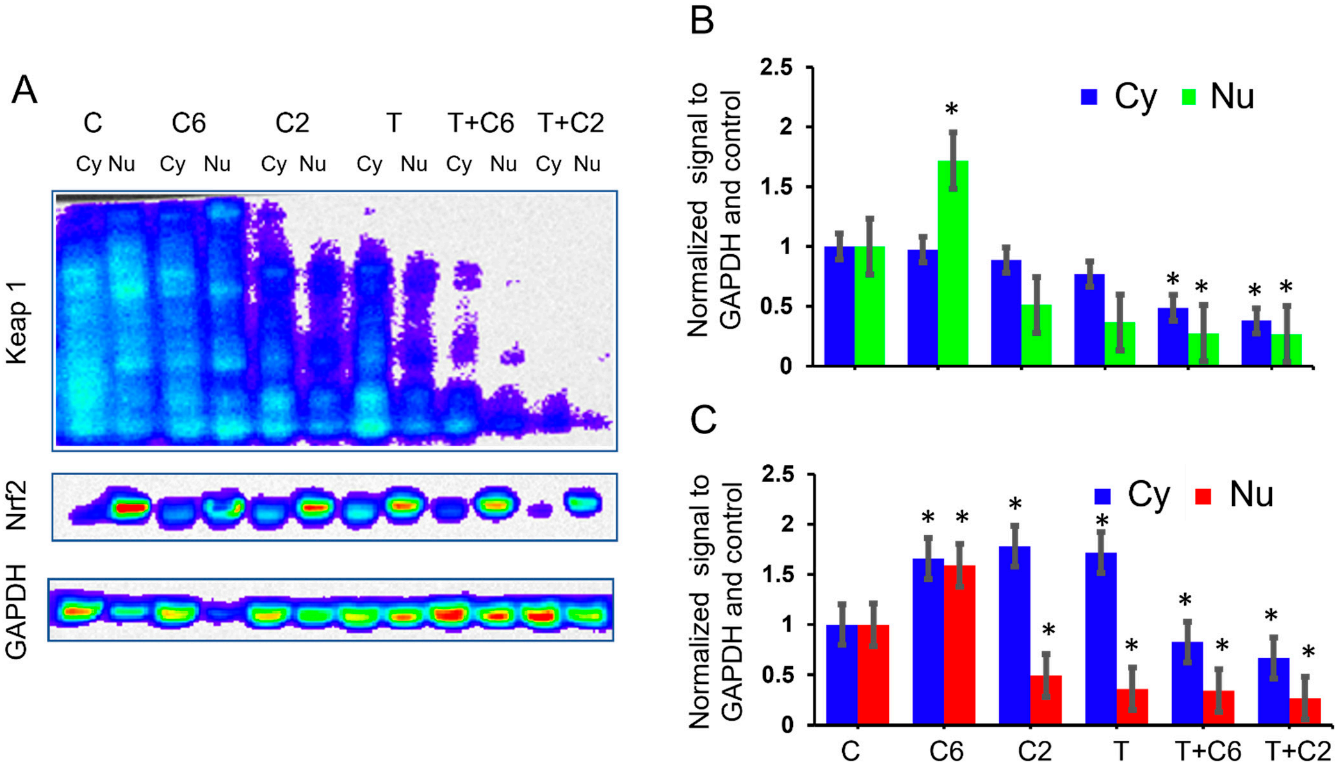

Figure S3. Mechanism of inhibition of Nrf2 in response to treatment with CET-CH-2 and CET-CH-6 in combination with TMZ. (**A**) Western blotting images of MDA-MB-231 cells treated with CET-CH-2, CET-CH-6, TMZ (T) or combination of them for 24 hours. C is control cells. (**B**) Quantitative plot showing the ratio of Keap1 expression in cytoplasm to nucleus. (**C**) Quantitative plot showing the ratio of Nrf2 expression in cytoplasm to nucleus. Protein expressions are measured in cytoplasm (Cy) and nucleus (Nu). In (**B**) and (**C**) the plots are normalized to control Cy and control Nu, respectively. C: control, C2: CET-CH-2, C6: CET-CH-6, and T: TMZ. Expression of Nrf2 and Keap1 are compared to the respective control (**C**) expression in each cell line is compared to the control signal in the corresponding site (i.e., Cytoplasm or nucleus), and * represents p-value < 0.05.

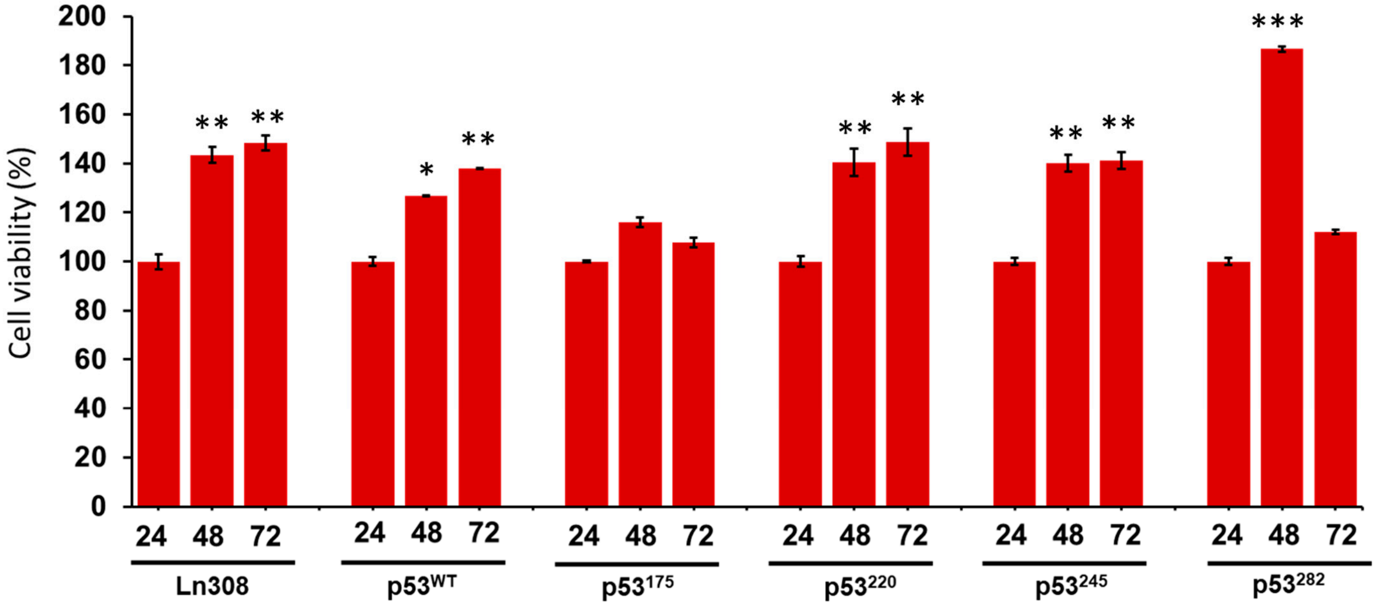

Figure S4. Proliferation of LN308 with different p53 mutations at 24, 48 and 72 hours post seeding. Cells were seeded in 96-well plate in triplicates and the proliferation of the cells were measured at

different time points by MTT assay. The results are normalized to 24 hours after seeding. Error bars represent Mean ± SD. *, **, *** represent p-value < 0.05, < 0.01, < 0.001, respectively

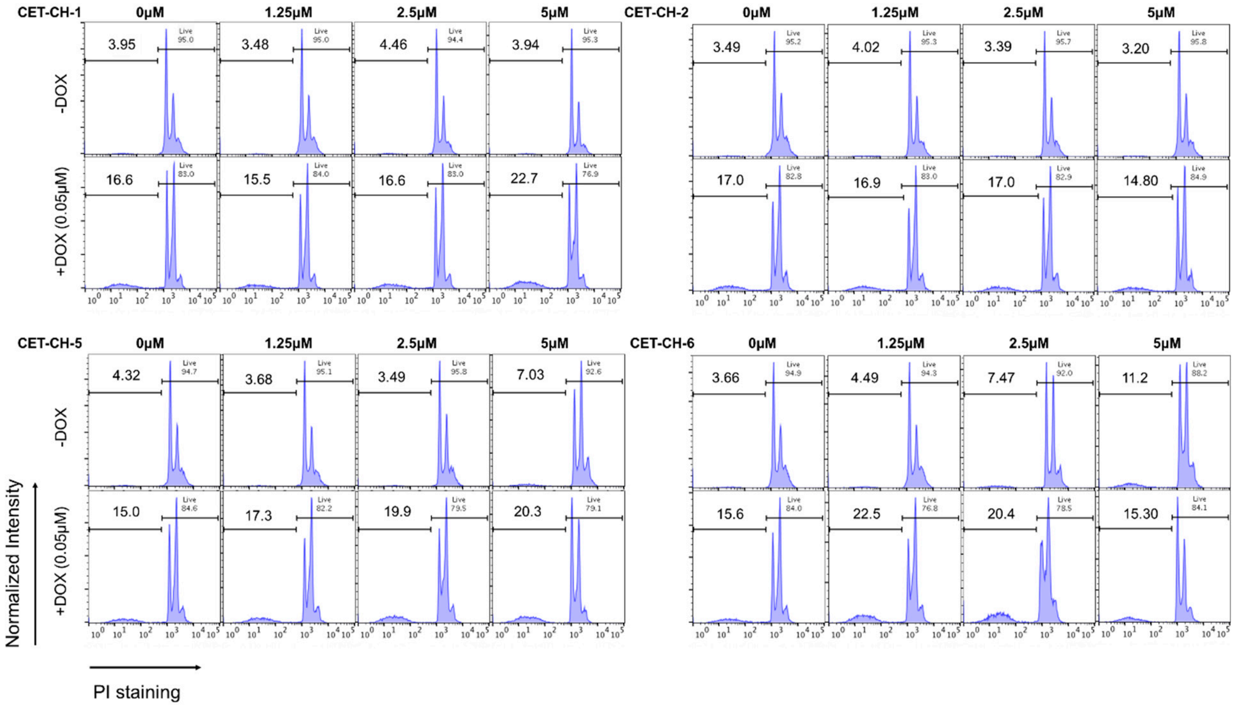

Figure S5. Differential therapeutic response of U87-MG-p53^{wt} to chemotherapy (DOX). Cells were treated in the presence of two Nrf2 activators (CET-CH-1 and CET-CH-2) and two Nrf2 inhibitors (CET-CH-5 and CET-CH-6) assessed by flow cytometry analysis for the induced apoptotic and live cell population. Cells were fixed using 70% ethanol, and the population of cells with low PI staining signal due to fragmented DNA are considered as late apoptotic population.

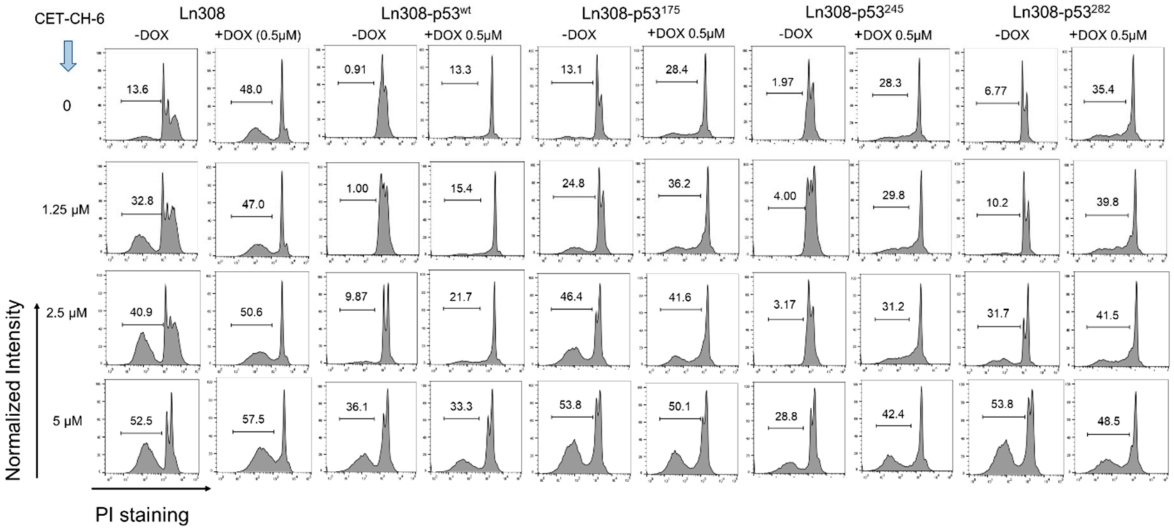

Figure S6. Differential therapeutic response of LN308 cells engineered to stably express different p53 mutations in response to the chemotherapy (DOX) treatment (0.5 μM) in the presence of different doses of CET-CH-6. Cells were treated in the presence of CET-CH-6 as assessed by flow cytometry analysis for the induced apoptotic population. Cells were fixed using 70% ethanol, and the population of cells with low PI staining signal due to fragmented DNA are considered as late apoptotic population. Numbers show the percentage of cells with fragmented DNA.

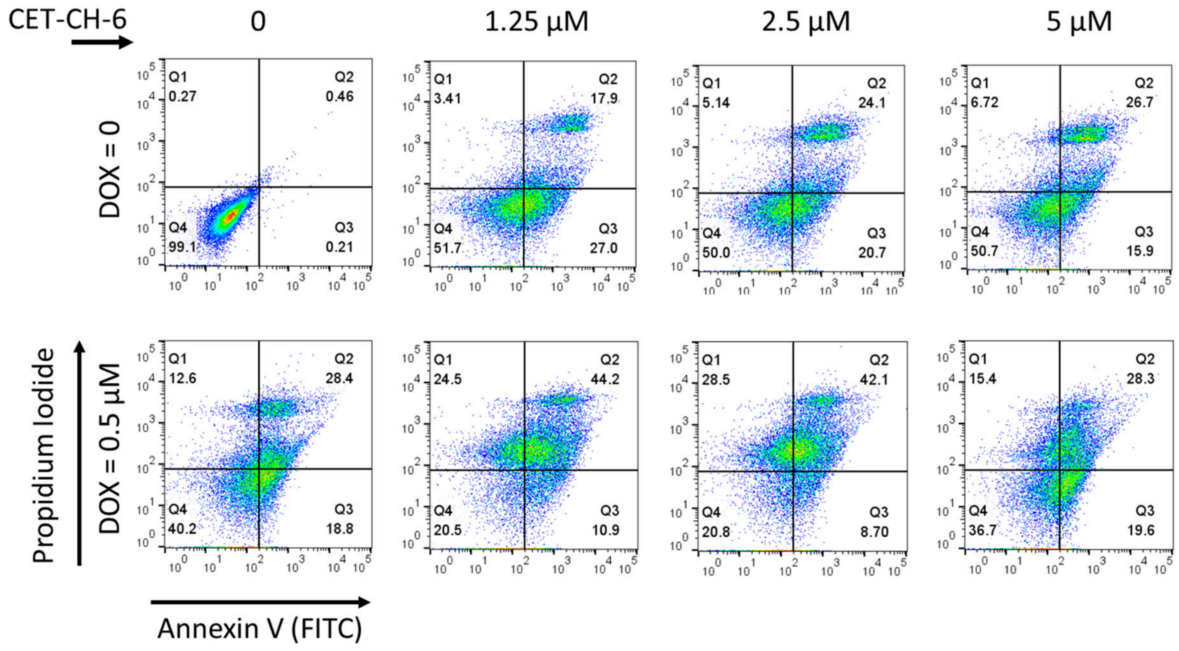

Figure S7. Annexin-V/PI staining assay to show differential therapeutic response of LN308-p53 Null cells to chemotherapy (DOX). Cells were treated in the presence of CET-CH-6 as assessed by flow cytometry analysis for the induced apoptotic population.

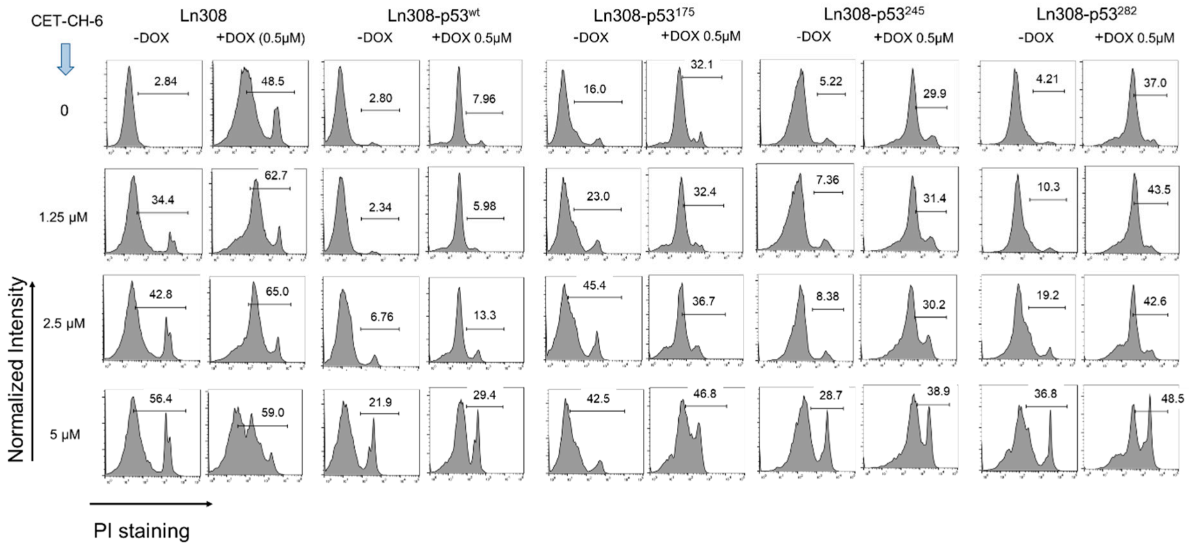

Figure S8. Annexin-V/ PI staining assay to show differential therapeutic response of LN308 cells stably expressing different p53 mutations to chemotherapy (DOX). Cells were treated in the presence of CET-CH-6 and assessed by flow cytometry analysis for the induced apoptotic population. Numbers show the percentage of apoptotic cells.

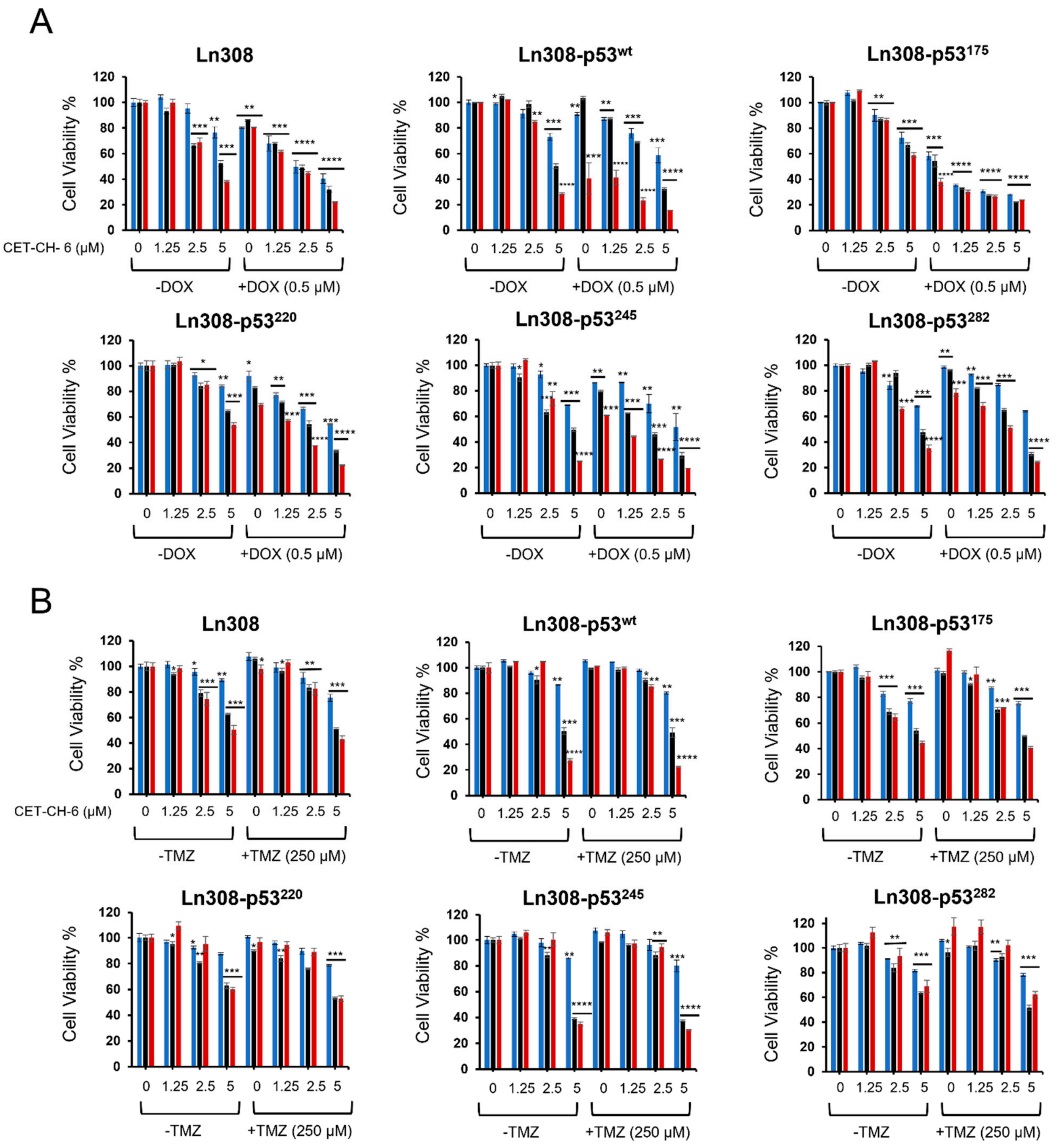

Figure S9. MTT assay result shows that co-treatment of CET-CH-6 with DOX and TMZ inhibits cell growth in LN308 cell lines with different p53 variants. Differential therapeutic responses of LN308 cells stably expressing different p53 variants as assessed using MTT in response to co-treatment with (**A**) CET-CH-6 and DOX, and (**B**) CET-CH-6 and TMZ. Each sample was normalized to the untreated control at the corresponding time. Experiments were performed in triplicate wells, and error bars represent mean ± SD. *, **, ***, **** represent p-value < 0.05, < 0.01, < 0.001, < 0.0001, respectively. Each sample was tested against the untreated control at the corresponding time. Blue, black and red bars represent assay results collected at 24, 48 and 72 h after treatment, respectively.

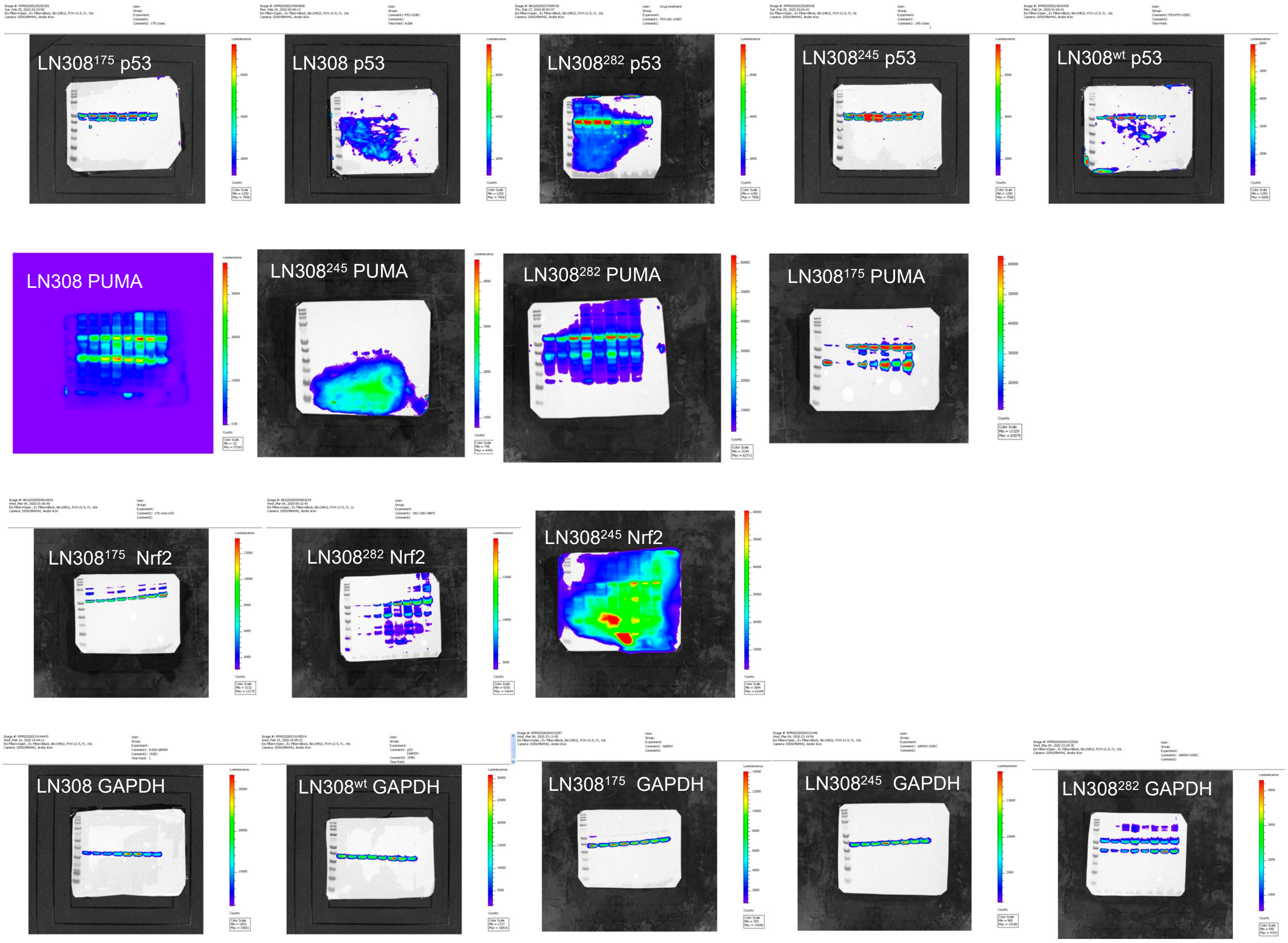

Figure S10. Whole western blotting membranes to show the location of each band and the intensities for Figure 7 in the manuscript.

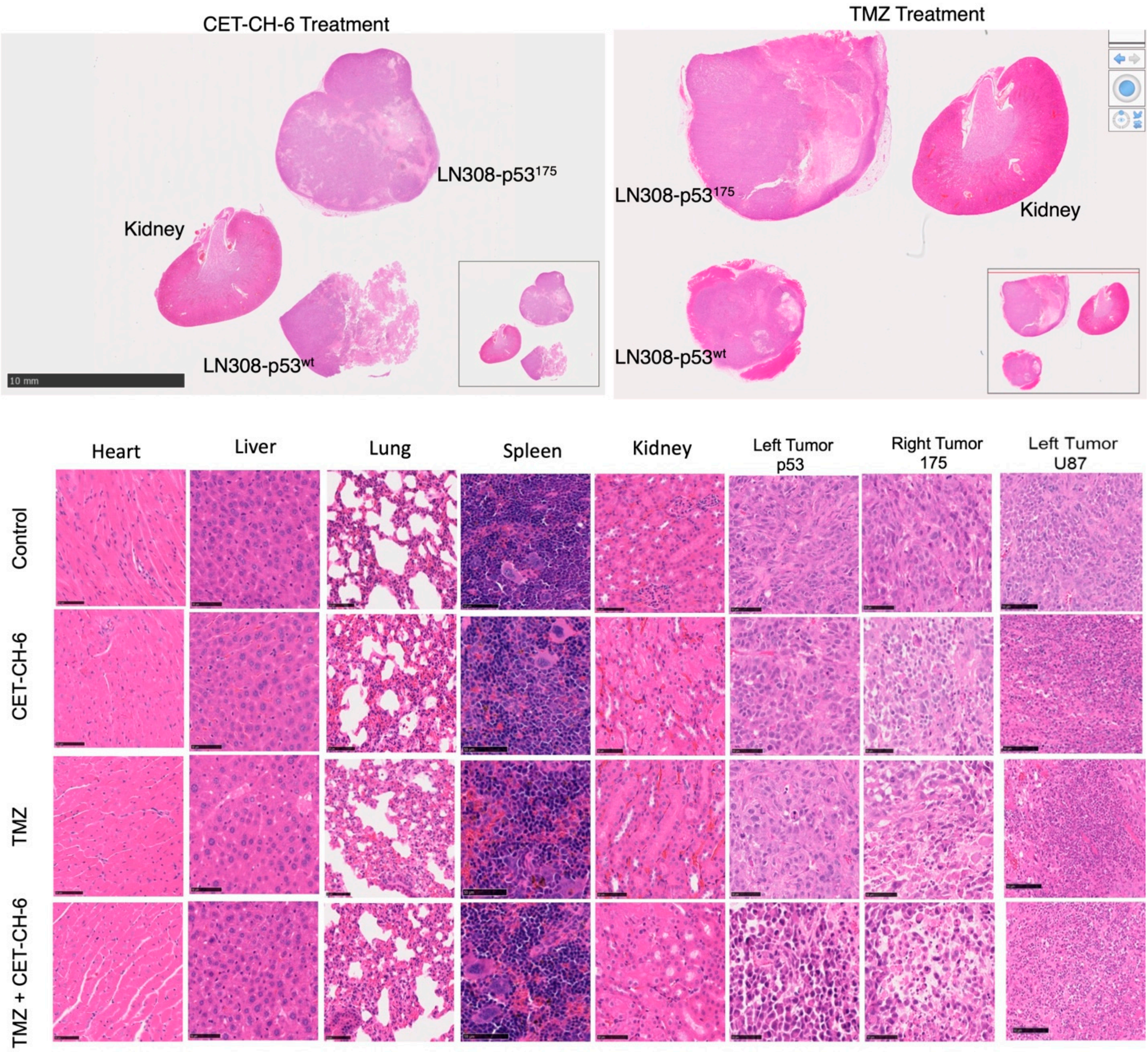

Figure S11. H&E staining of different organs and tumors. NSG mice bearing U87-MG, LN308-p53175 and U87-MG-p53wt cells. The bars represent 50 μm.
